# Platelet P-selectin triggers rapid surface exposure of tissue factor in monocytes

**DOI:** 10.1038/s41598-019-49635-7

**Published:** 2019-09-16

**Authors:** Ivelin I. Ivanov, Bonita H. R. Apta, Arkadiusz M. Bonna, Matthew T. Harper

**Affiliations:** 1 Department of Pharmacology, University of Cambridge, Cambridge, United Kingdom; 2 Department of Biochemistry, University of Cambridge, Cambridge, United Kingdom

**Keywords:** Mechanisms of disease, Platelets

## Abstract

Tissue factor (TF) plays a central role in haemostasis and thrombosis. Following vascular damage, vessel wall TF initiates the extrinsic coagulation cascade. TF can also be exposed by monocytes. Inflammatory or infectious stimuli trigger synthesis of new TF protein by monocytes over the course of hours. It has also been suggested that monocytes can expose TF within minutes when stimulated by activated platelets. Here, we have confirmed that monocytes rapidly expose TF in whole blood and further demonstrate that platelet P-selectin exposure is necessary and sufficient. Monocyte TF exposure increased within five minutes in response to platelet activation by PAR1-AP, PAR4-AP or CRP-XL. PAR1-AP did not trigger TF exposure on isolated monocytes unless platelets were also present. In whole blood, PAR1-AP-triggered TF exposure required P-selectin and PGSL-1. In isolated monocytes, although soluble recombinant P-selectin had no effect, P-selectin coupled to 2 µm beads triggered TF exposure. Cycloheximide did not affect rapid TF exposure, indicating that *de novo* protein synthesis was not required. These data show that P-selectin on activated platelets rapidly triggers TF exposure on monocytes. This may represent a mechanism by which platelets and monocytes rapidly contribute to intravascular coagulation.

## Introduction

Tissue factor (TF; CD142) plays a central role in haemostasis and thrombosis^1,2^. TF is a transmembrane glycoprotein constitutively expressed in the adventitia of normal blood vessels^2^. In the absence of vascular injury, TF is not exposed to flowing blood. Vascular damage allows plasma FVIIa to bind to TF, initiating the extrinsic pathway of coagulation. The TF:FVIIa complex activates FX; FXa is the central component of the prothrombinase complex, leading to formation of thrombin^3,4^. Thrombin cleaves plasma fibrinogen to release fibrin, and activates platelets through protease-activated receptors (PARs)^3,5^. Exposure of TF to blood following vascular injury is therefore a key trigger for coagulation and haemostasis.

TF can also be exposed to blood in pathological circumstances. Atherosclerotic plaques contain high levels of TF that is exposed on plaque rupture^6^. Subsequent TF-triggered coagulation contributes to arterial thrombosis and acute coronary syndrome^7,8^. Blood-borne TF may also contribute to intravascular coagulation and thrombosis^9,10^. Monocytes are a central source of blood-borne TF following inflammatory or infectious stimuli^4,11,12^. Bacterial lipopolysaccharide (LPS), for example, induces increased expression of TF in monocytes^12^. This is dependent on synthesis of new TF protein^13^. TF on the monocyte surface is often shed in extracellular vesicles known as microparticles^14^. These circulating, TF-positive microparticles are likely to be the major source of blood-borne TF. However, this increase in TF gene expression occurs over hours^12^, so although on-going TF gene expression in monocytes may contribute to increased thrombosis risk in inflammatory or infectious disease states, it may be less likely to contribute to rapid arterial thrombosis if it has not previously been triggered.

However, there have been isolated reports that monocytes can expose TF on their surface much more rapidly, for example, within ten minutes (e.g.^15^). This has been observed in whole blood, where cells other than monocytes are also present. It has been proposed that blood platelets, when activated, bind to monocytes. In response, monocytes expose TF on their surface. This occurs without an increase in TF mRNA, suggesting that it is not dependent on synthesis of new TF protein^15^. In our study, we have confirmed that monocytes rapidly expose TF in whole blood. Furthermore, we used isolated cells and recombinant P-selectin to demonstrate that platelet P-selectin exposure is both necessary and sufficient.

## Results

### Monocytes rapidly expose TF in response to platelet activators in whole blood

**Figure 1:**
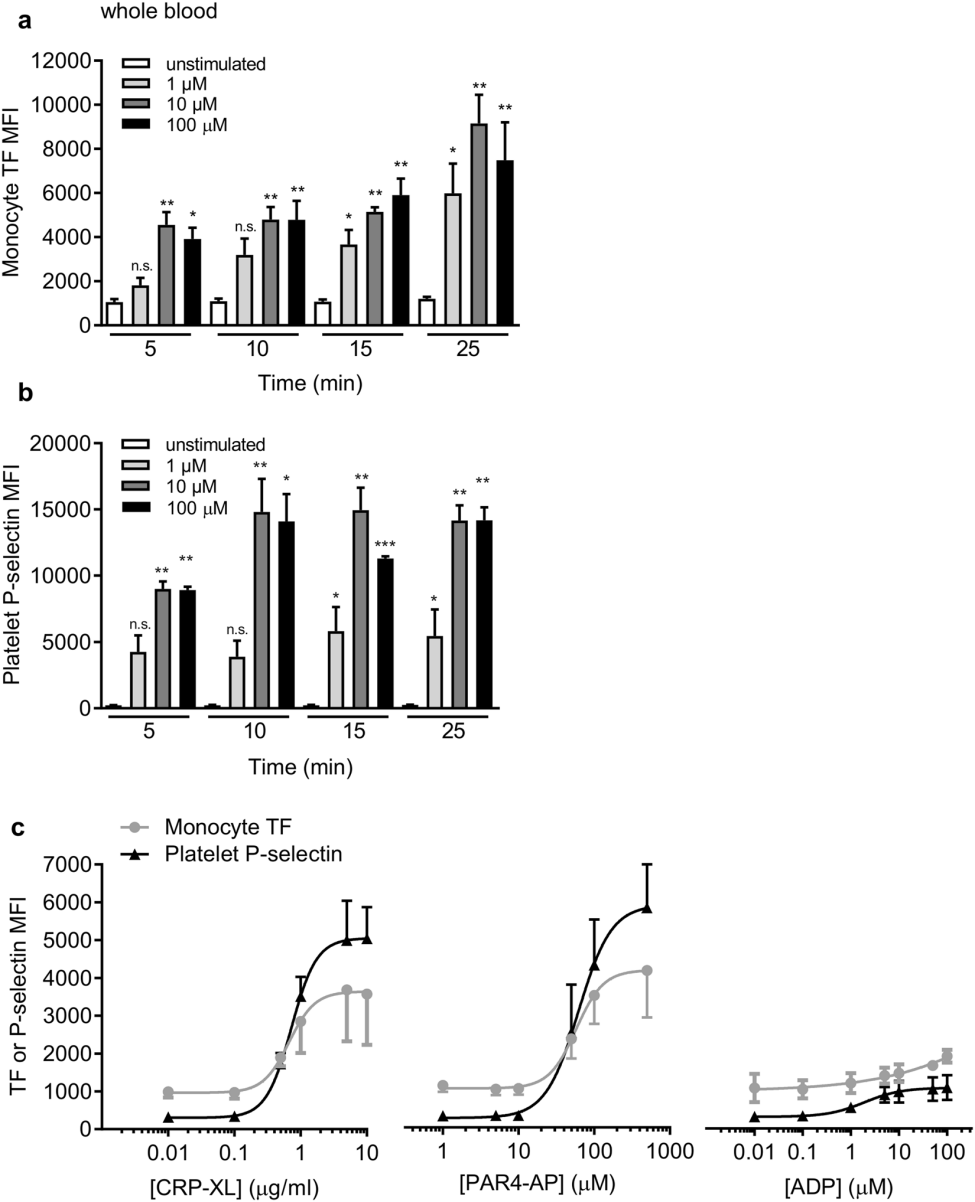
Platelet activators rapidly trigger monocyte TF exposure in whole blood. (**a**,**b**) PPACK-anticoagulated human whole blood was stimulated with PAR1-AP (1–100 µM; 5–25 mins) prior to staining with conjugated primary antibodies. Median fluorescence intensity (MFI) is shown for TF on CD14 + monocytes (**a**) and P-selectin on CD41a + platelets (**b**) (mean + S.E.M.; n = 5; n.s. not significant p > 0.05; *p < 0.05; **p < 0.01 compared to unstimulated at the same timepoint; Friedman’s non-parametric test with Dunn’s multiple comparison post-test). (**c**) Whole blood was stimulated with a range of concentrations of the platelet activators, CRP-XL, PAR4-AP, and ADP, for 10 minutes. Monocyte TF and platelet P-selectin are shown on the same axes for comparison of concentration-dependency of their effects.

PPACK-anticoagulated blood was stimulated with the PAR1 agonist, PAR1-AP (SFLLRN-amide), and analysed by flow cytometry. Within 5 minutes of stimulation, we detected TF antigen on the surface of CD14^+^ monocytes in a concentration-dependent manner (Fig. 1a). In parallel, CD41a^+^ platelets exposed P-selectin, indicating α-granule secretion (Fig. 1b). Similar results were seen with two different platelet activators, PAR4-AP (AYPGKF-amide), a selective PAR4 activating peptide, and cross-linked collagen-related peptide (CRP-XL), a selective GPVI agonist (Fig. 1c). Both activators rapidly increased platelet surface P-selectin and monocyte surface exposure of TF. In contrast, ADP only weakly activated platelets under these conditions (shown by the relatively small increase in P-selectin), and only weakly stimulated monocyte surface exposure of TF. With all activators, the concentration-dependence of monocyte TF exposure and platelet P-selectin exposure was similar. For CRP-XL, the EC_50_ for TF exposure was 0.67 µg/ml and the EC_50_ for P-selectin exposure was 0.73 µg/ml. For PAR4-AP, the EC_50_ was 58 µM and 62 µM for TF and P-selectin exposure, respectively. The TF on the monocyte surface was detected by three different antibodies that detect different epitopes of TF (Supplementary Fig. 1), suggesting that this binding does indeed represent TF antigen.

**Figure 2:**
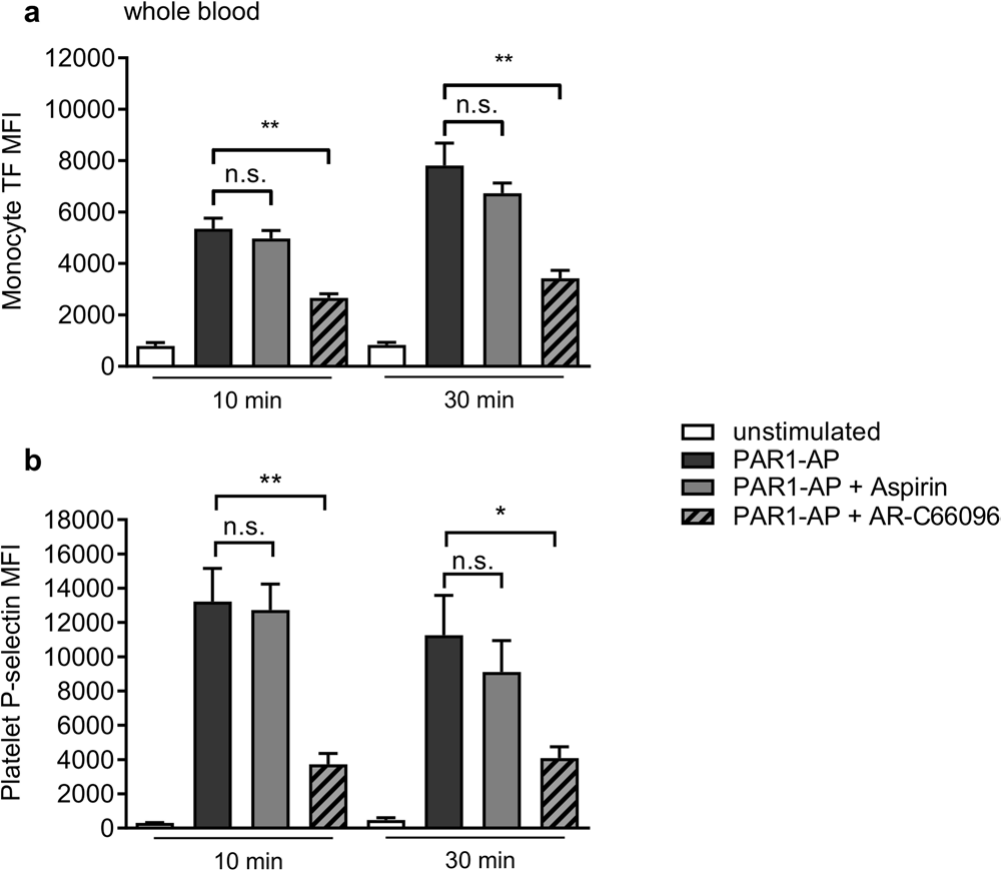
P2Y_12_ inhibition reduces monocyte TF and platelet P-selectin exposure. Whole blood was treated with aspirin (100 µM), the P2Y_12_ antagonist, AR-C69906 (10 µM), or their solvents as control, for 10 min prior to stimulation with PAR1-AP (10 µM). Data are mean + S.E.M. (n = 5; n.s. not significant; *p < 0.05; **p < 0.01 for indicated comparison).

Aspirin and P2Y_12_ antagonists are used clinically to inhibit platelet activation^16^. Pre-treatment of whole blood *ex vivo* with aspirin (100 µM) had no effect on monocyte TF or platelet P-selectin exposure under these conditions (Fig. 2). In contrast, the P2Y_12_ antagonist, AR-C66096 (10 µM), reduced PAR1-AP-triggered surface TF exposure to 42.4 ± 3.8% (n = 5; p < 0.01) at 10 minutes of stimulation, and to 37.8 ± 2.2% (n = 5; p < 0.01) at 30 minutes. Platelet P-selectin exposure was also inhibited, consistent with previous reports^17^, suggesting that the reduction in TF may be a consequence of inhibited platelet activation.

### Platelets are required for rapid surface exposure of TF in monocytes

**Figure 3:**
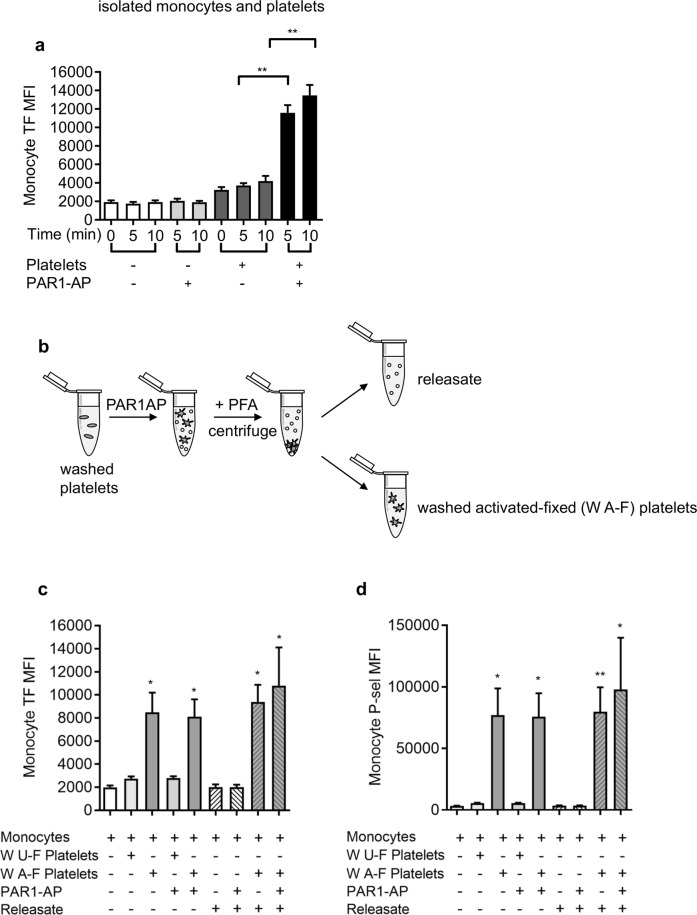
Platelets are necessary and sufficient for rapid monocyte TF exposure. (**a**) Isolated monocytes were treated with PAR1-AP (10 µM, 5–10 min) in the absence or presence of washed platelets. (n = 5; ***P < 0.001 for indicated comparison) (**b**) Washed platelets were stimulated with PAR1-AP, fixed with paraformaldehyde (PFA) then collected by centrifugation to separate the *releasate* (supernatant) and *washed activated-fixed* (W A-F) platelets (pellet). As a control, some platelets left unstimulated prior to fixation (*washed unactivated-fixed*, W U-F) (**c**,**d**) Isolated monocytes were incubated with washed unactivated-fixed (W U-F), washed activated-fixed platelets (W A-F) and/or releasate fractions for 5 min. Monocyte TF exposure (**c**) and mono**c**yte-bound P-selectin (**d**) are shown as mean + S.E.M. (n = 5; *p < 0.05; **p < 0.01 compared to monocytes alone; Friedman’s non-parametric test with Dunn’s multiple comparison post-test).

To investigate the role of platelets in the rapid surface exposure of TF in monocytes, we isolated monocytes and platelets from whole blood. Monocytes alone stimulated with PAR1-AP did not expose TF (Fig. 3a), indicating that this agonist is not acting directly on the monocytes. Similarly, TF was not detected on the surface of platelets alone when stimulated with PAR1-AP. In contrast, when monocytes and platelets were combined, TF was detected on CD14^+^ monocytes following stimulation with PAR1-AP (Fig. 3a). Together, these data indicate that activated platelets are required for the rapid exposure of TF.

We used two approaches to determine whether a soluble mediator released by platelets might contribute to monocyte TF exposure. First, platelets were stimulated with PAR1-AP, fixed with paraformaldehyde (PFA), then isolated by centrifugation to remove any soluble released factors (‘washed, activated-fixed’, W A-F; Fig. 3b). As a control, some platelets were left unstimulated prior to being fixed (‘washed unactivated-fixed’, W U-F). When activated-fixed platelets were added to isolated monocytes, TF was exposed to a similar level to that seen previously (Fig. 3c). Representive flow cytometry density plots are shown in SFig. 3. PAR1-AP had no further effect. Second, the supernatant (‘releasate’; Fig. 3b), which contains soluble factors released by activated platelets, was added to isolated monocytes. No surface exposure of TF was detected, even when PAR1-AP was also added (in case a soluble factor was acting in synergy with PAR1-AP). Furthermore, the releasate (and PAR1-AP) had no further effect on monocyte TF in addition to activated, fixed platelets (Fig. 3c). These results indicate that the signals from platelets that trigger monocyte TF exposure are mediated by contact between activated platelets and monocytes. Notably, activated-fixed platelets provided P-selectin to monocytes, whereas the releasate did not (Fig. 3d). This suggests that the stimulation did not trigger release of P-selectin-positive microparticles that then adhere to monocytes. In addition, these data also show that stimulated monocytes themselves do not expose P-selectin, since no P-selectin was detected in the absence of activated platelets.

### P-selectin and PGSL-1 are required for rapid surface exposure of TF in monocytes

**Figure 4:**
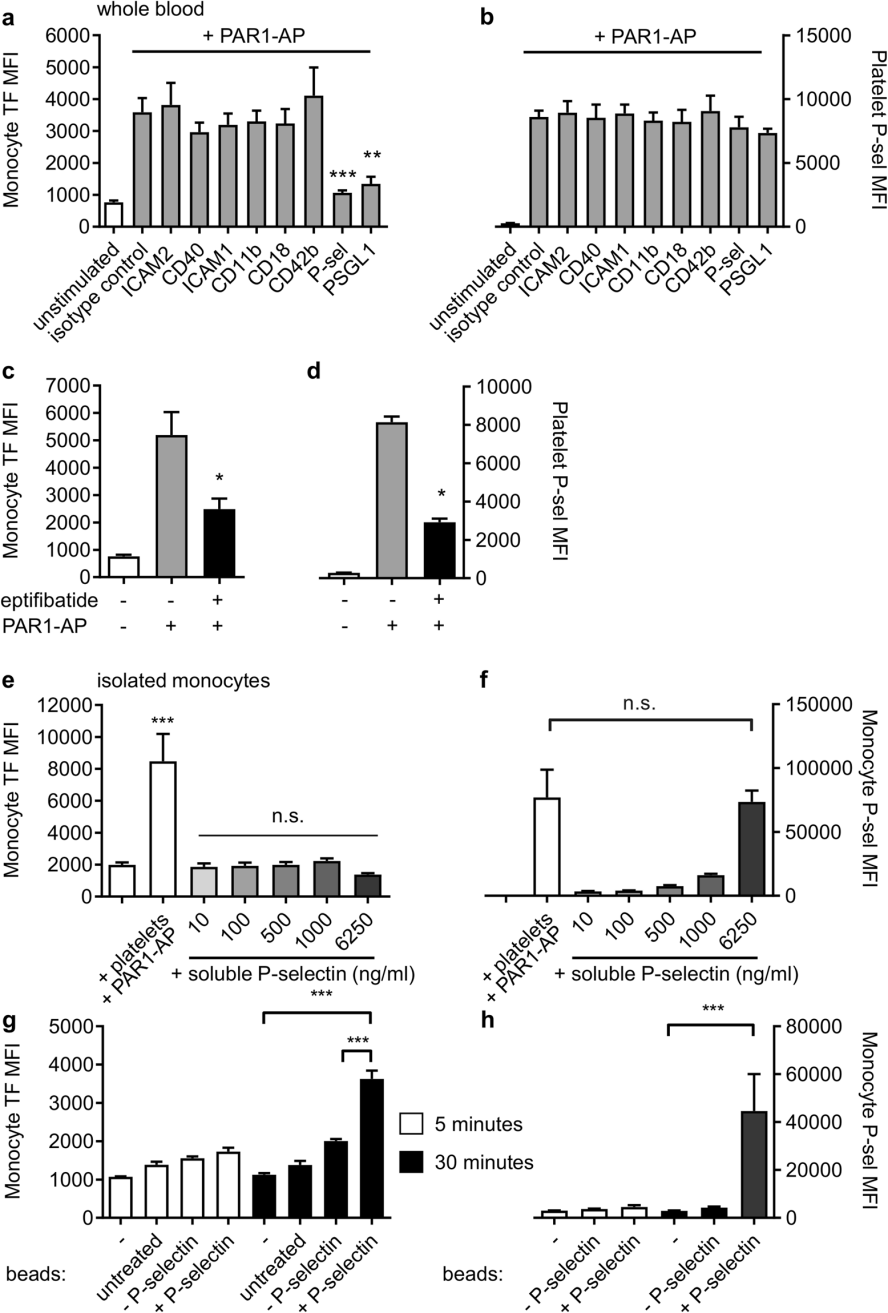
P-selectin is necessary and sufficient for rapid monocyte TF exposure. PPACK-anticoagulated whole blood was treated with blocking antibodies against ICAM1, ICAM2, CD40, CD11b, CD18, CD42b, P-selectin and PSGL1 (**a-b**) or the α_IIb_β_3_ antagonist, eptifibatide (10 µM) before stimulation with PAR1-AP (10 μM, 10 min; **c-d**). Monocyte TF (**a**, **c**) and platelet P-selectin (**b**, **d**) were detected by flow cytometry (mean + S.E.M; n = 3–6; *p < 0.05; **p < 0.01; ***p < 0.001 compared to PAR1-AP-stimulated samples in absence of inhibitor or blocking antibody; one-way ANOVA with Dunnett’s multiple comparisons post-test). (**e**,**f**) Isolated monocytes were stimulated with platelets and PAR1-AP (as positive control) or with soluble recombinant P-selectin at the concentrations indicated for 30 min (**e**,**f**) or with control beads or P-selectin-coated beads (**g**,**h**). Monocyte TF exposure and monocyte-bound P-selectin are shown in (**e**,**g**) and (**f**,**h**), respectively. (***p < 0.001 for indicated comparison; one-way ANOVA with Dunnett’s multiple comparison post-test; in **e**, n.s. indicate not significantly different from monocytes alone).

Blocking antibodies were used to determine the contribution of adhesion receptors to platelet-monocyte binding and TF exposure. Neither platelet-monocyte nor TF exposure was affected by antibodies that block ICAM1, ICAM2, CD40, CD11b, CD18, or CD42b. In contrast, platelet-monocyte binding and TF exposure was almost completely inhibited by antibodies that block either P-selectin (found on the surface of stimulated platelets) or its receptor on monocytes, PGSL-1 (Fig. 4a,b). A small molecule antagonist of platelet α_IIb_β_3_, eptifibatide, did partially reduce platelet-monocyte binding and TF exposure (Fig. 4c; reduced to 46.1 ± 5.0%; n = 5; p < 0.05). However, under these conditions, eptifibatide also reduced platelet P-selectin exposure (Fig. 4d; reduced to 32.7 ± 1.9%; n = 5; p < 0.05).

### Recombinant P-selectin triggers monocyte TF surface exposure when coupled to beads

Since P-selectin was necessary for rapid surface exposure of TF, we investigated whether P-selectin alone was sufficient. Monocytes were treated with recombinant human P-selectin (10-6250 ng/ml). Although soluble P-selectin rapidly bound to monocytes, it did not trigger surface exposure of TF after 5 or 30 minutes (Fig. 4e), despite P-selectin binding to monocytes to a level similar to that provided by activated platelets bound to monocytes (Fig. 4f). In contrast, when the same recombinant P-selectin was coupled to 2 µm beads, it could trigger surface expression of TF (Fig. 4g). This was relatively slow (compared to stimulated platelets), occurring after 30 minutes but not after 5 minutes. However, the P-selectin beads also took the same time to bind to monocytes (Fig. 4h). Untreated beads, and beads that had been chemically activated but not coupled to P-selectin, did not trigger surface expression of TF.

### Rapid monocyte TF surface exposure does not require protein synthesis

**Figure 5:**
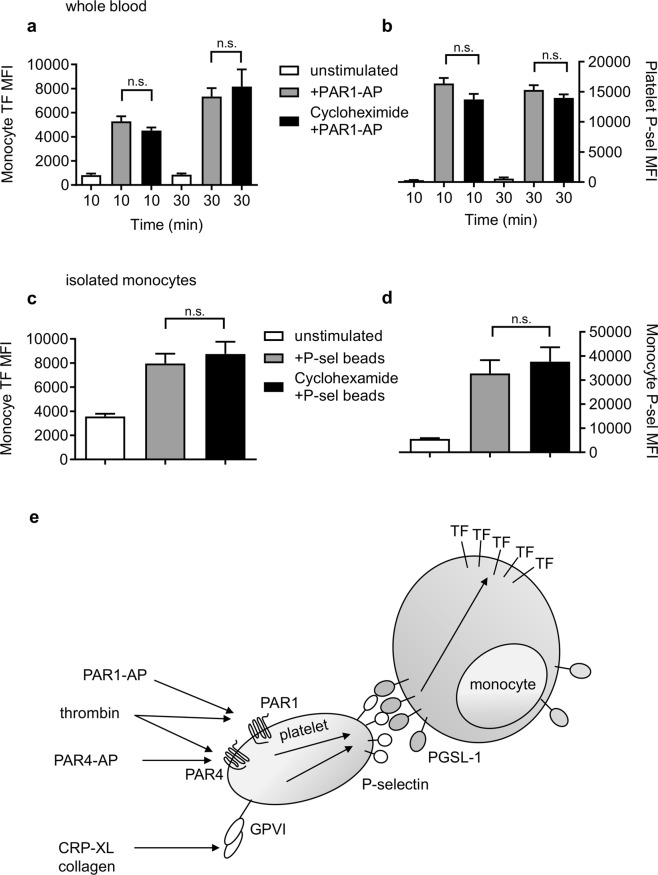
Rapid monocyte TF exposure does not require protein synthesis. PPACK-anticoagulated blood (**a**,**b**) or isolated monocytes (**c**,**d**) were treated with cycloheximide (100 µM) or vehicle control. Whole blood was stimulated with PAR1-AP for the indicated times and isolated monocytes were stimulated with P-selectin-coated beads for 30 min. (n.s. indicates no significant difference for indicated comparison; one-way ANOVA with Tukey’s multiple comparison post-test.) (**e**) Summary of proposed rapid platelet-dependent exposure of monocyte TF. Platelet activators trigger α-granule secretion leading to P-selectin exposure on the platelet surface. P-selectin interacts with monocyte PGSL-1, rapidly triggering TF exposure without new protein expression.

In response to LPS, monocytes synthesise new TF protein within a few hours. To test whether new protein synthesis was involved in the much more rapid surface exposure of TF, whole blood was treated with cycloheximide (100 µM), an inhibitor of translation. PAR1-AP was still able to trigger rapid TF exposure in the presence of cycloheximide (Fig. 5a,b). Similarly, isolated monocytes treated with P-selectin-coupled beads also showed surface expression of TF in the presence of cycloheximide (Fig. 5c,d). This indicates that rapid surface exposure of TF in monocytes is not dependent on protein synthesis.

## Discussion

Monocytes are a major source of intravascular TF. In this study, we demonstrate that activated platelets can rapidly trigger surface exposure of TF in monocytes through P-selectin interaction with monocyte PSGL1 (Fig. 5e). This surface exposure of TF occurs within minutes and does not depend on synthesis of new TF protein. This represents a mechanism by which platelets and monocytes might rapidly contribute to intravascular coagulation.

Platelet activation by a range of receptor agonists rapidly led to surface exposure of TF by monocytes in whole blood. TF antigen was readily detectable in these aggregates with three different anti-TF antibodies. Although similar observations have been reported by Lindmark *et al*.^15^, which adds further confidence to our findings, that previous report was unable to determine whether platelets were essential to this response, or whether there may be direct stimulation of the monocytes (or perhaps other cells such as lymphocytes) in the whole blood assay. However, our results also demonstrate that platelets are essential to this response. Isolated monocytes did not expose TF in response to the PAR1 stimulation unless platelets were also present. Moreover, the experiments with activated, fixed platelets demonstrate that activated platelets trigger monocyte TF surface exposure even when the initial platelet activator has been removed (and so is not acting on the monocytes). These experiments demonstrate that platelets are both necessary and sufficient for rapid surface exposure of TF in monocytes.

The signal between activated platelets and monocytes appears to be entirely transduced by the P-selectin – PSGL1 interaction in our system. Activated, fixed then washed platelets were capable of fully triggering the response, whereas the platelet releasate had no effect. This suggests that a soluble factor secreted by platelets is not responsible. Monocyte TF exposure was entirely inhibited by blocking antibodies to P-selectin or to PSGL-1, showing these two adhesion molecules to be necessary. Moreover, P-selectin is sufficient to trigger the response in monocytes, as shown by the experiments with exogenous recombinant P-selectin. Interestingly, soluble monomeric P-selectin did not trigger monocyte TF exposure, despite rapidly coating the monocyte surface with P-selectin. P-selectin in platelet α-granules is found as a homodimer and is exposed on the platelet surface as a dimer following granule exocytosis^18^. When P-selectin was coupled to 2 µm beads it could trigger TF exposure. This suggests that clustering of PSGL-1 by P-selectin may contribute to intracellular signalling in monocytes and that the clustering of P-selectin into dimers or oligomers is important for its signalling function in platelets. Similarly, Panicker *et al*.^19^ showed that dimeric P-selectin binds to PGSL-1 with greater avidity than monomeric P-selectin, and that dimeric or oligomeric soluble P-selectin was required for inducing leukocyte signalling. It also explains the apparent discrepancy between our findings, and those of Celi *et al*.^20^ and del Conde *et al*.^21^. Although these investigators clearly showed that soluble P-selectin is capable of increasing monocyte TF over the course of several hours by increasing protein synthesis, they found no rapid effect. One possible explanation is that they used soluble monomeric P-selectin, which induced weak, slow signalling, rather than a form that might be able to cluster counter-receptors such as PSGL-1. It also suggests that circulating soluble monomeric P-selectin, which is a potential biomarker for cardiovascular disease^22^, might be largely ineffective, whereas cells that expose P-selectin, such as platelets, may be more effective.

Although the P-selectin-coupled beads were capable of triggering monocyte TF surface exposure, this was slower than seen with platelet activation either in whole blood or with isolated cells. We suspect that this is an artefact of our experimental conditions. During our coupling reaction, the carboxylic acid on the beads is linked to a primary amine in the recombinant P-selectin. However, there are many primary amines available in P-selectin. Not all the protein molecules will be in a productive position or orientation. This will lower the overall activity. In addition, in preliminary experiments, we found that the coupling reagent (EDAC/carbodiimide) could induce the beads to adhere to monocytes in the absence of P-selectin (presumably to primary amines in proteins on the monocyte surface); untreated beads did not adhere. The addition of BSA to quench reactive coupling reagent prevented P-selectin-independent binding, but it did slow the binding of P-selectin-coupled beads to the monocytes, and so slightly delayed the exposure of TF. Notably, this slightly delayed TF exposure was also independent of protein synthesis (see below).

Another possibility is that there are signals from activated platelets in addition to P-selectin. As discussed above, there does not appear to be a soluble secreted factor involved. However, there may be an additional platelet surface molecule involved. We have excluded several adhesion receptors on platelets or monocytes through use of blocking antibodies. If there is an additional signal on the surface of activated platelets, it does not involve CD40 or CD42 on platelets, or ICAM-1, ICAM-2, CD11b or CD18 on monocytes. The small molecule α_IIb_β_3_ antagonist, eptifibatide, did reduce monocyte surface TF, but this was also associated with inhibition of platelet P-selectin exposure. Although it could indicate an additional role for α_IIb_β_3_ in the platelet-monocyte interaction, it may also reflect the role of integrin outside-in signalling in amplifying platelet activation^23,24^. Similarly, inhibition of P2Y_12_ also reduced monocyte TF exposure in parallel with inhibition of platelet P-selectin exposure, and this likely reflects the role of P2Y_12_ in enhancing α-granule secretion^17^. In contrast, under our conditions, aspirin had no effect on monocyte TF exposure, consistent with a lack of effect on platelet P-selectin exposure. (Although aspirin is used as an anti-thrombotic in patients, its effect on platelet activation *in vitro* is often relatively weak and depends on the primary activator being used (see, for example, Blair *et al*.^25^).

Rapid platelet-dependent exposure of monocyte TF did not require *de novo* protein synthesis, as it was not affected by cycloheximide. Similarly, Lindmark *et al*.^15^ found no increase in TF mRNA. This suggests that TF is already expressed in unactivated monocytes, providing a small pool of TF that can be rapidly mobilised. Intracellular TF expression in unstimulated monocytes is likely to be low, and is reported to be not present by some groups, though it has been detected (at a low level) in some reports (e.g.^26^). Any constitutive TF expression in monocytes would need to be maintained in an intracellular compartment and/or in an inactive form to prevent intravascular coagulation. In macrophages, constitutive internalization of surface TF occurs in a dynamin- and integrin α4β1-dependent manner^27^. A similar mechanism may prevent surface TF exposure in unstimulated monocytes. TF activity may also be controlled by ‘encrypting’ the TF such that it does not activate FX. In addition to surface exposure, decryption of TF may be required for its activity^28,29^. In preliminary experiments, we have been unable to reliably detect tenase activity in isolated monocytes following rapid exposure of TF, suggesting that additional signals may be required for TF activity^30,31^.

We propose that monocytes could be recruited to sites of arterial injury, such as atherosclerotic plaque rupture or erosion, by adherent, activated platelets^32–34^, and that platelet P-selectin would trigger monocyte TF exposure. If the TF is active – possibly with an additional decryption signal – then activation of the extrinsic coagulation cascade may contribute to the growth of an intra-luminal thrombus that may occlude the artery. Anti-platelet drugs, such as aspirin and P2Y_12_ antagonists, are important treatments for the prevention of arterial thrombosis. However, all currently-approved anti-platelet drugs are associated with increased bleeding risk, owing to platelets’ role in haemostasis^16^. If rapid monocyte TF exposure contributes to thrombosis, then it may provide new target for treatment. This is consistent with a variety of *in vivo* thrombosis studies. Inhibition of P-selectin reduced arterial thrombosis^35,36^ and was associated with fewer leukocytes within thrombi^35^ in mice. P-selectin and PGSL-1 were required for TF and fibrin accumulation in a laser-induced arteriolar thrombosis murine model (although in this model it is likely to be TF-bearing microparticles from monocytes rather than monocytes themselves that promote fibrin formation)^37^. In a baboon arteriovenous shunt model, a blocking antibody to platelet P-selectin inhibited leukocyte accumulation and fibrin formation^38^. Although more experimental validation is needed, a role for rapid, P-selectin-dependent monocyte TF exposure in thrombosis is consistent with previous reports and is a potential target for anti-thrombotic therapy. Conversely, inhibition of platelet P-selectin exposure by current antiplatelet drugs such as P2Y_12_ antagonists may contribute to their antithrombotic benefit.

## Methods

### Blood collection

Use of human blood from healthy volunteers was approved by the Human Biology Research Ethics Committee, University of Cambridge. The volunteers gave fully-informed, written consent in accordance with the Declaration of Helsinki. The volunteers did not take any medications, including non-steroidal anti-inflammatory drugs, antihistamines, and antibiotics, for at least 14 days prior to blood acquisition. Different anticoagulants were used depending on the assay, as noted below.

### Stimulation of whole blood

For whole blood experiments, blood was collected in Sample Collection/Anticoagulant Tubes containing the anticoagulant lyophilised Phe-Pro-Arg-chloromethylketone (PPACK, final concentration 75 µM, Haematologic Technologies, VT, USA). 50 µl whole blood was stimulated with agonist for defined times, stained directly conjugated primary antibodies for 5 minutes (see below), then diluted with 350 µl 1xFix/Lyse solution (eBioscience). Samples were kept on ice in the dark until analysis by flow cytometry.

### Platelet isolation

Whole blood was collected in sodium citrate-containing Vacutainers (Becton Dickinson). Citrated blood was centrifuged (200 x g, 10 min, 30 °C) to obtain platelet-rich plasma (PRP). This was collected and diluted 1:1 with HBS-glucose (HEPES-buffered saline: 10 mM HEPES, 135 mM NaCl, 3 mM KCl, 0.34 mM NaH_2_PO_4_, 1 mM MgCl_2_.6H_2_O, pH 7.4; supplemented with 0.9 mg/ml D-glucose). PGE_1_ (prostaglandin E_1_, 100 nM) and apyrase (0.02 U/ml) were added to prevent platelet activation. PRP was centrifuged (600 × g, 10 min, 30 °C) and platelets resuspended in HBS-glucose. 2 mM CaCl_2_ was added immediately prior to stimulation.

### Monocyte isolation

EDTA-anticoagulated blood (1.8 mg/ml) was taken from the same respective donor as the platelets. OptiPrep density gradient (Sigma) was thoroughly mixed with the blood to increase its density. The mixture was layered under Histopaque 1077 (Sigma) in a 15 ml Falcon tube using a 15-cm blunt needle and a syringe. The blood was centrifuged (700 x g, 20 min, 20 °C). The PBMC band (at the top) was collected and the cells were washed with 0.15% BSA solution in PBS pH 7.4 and centrifuged (200 x g, 10 min, 20 °C). The cells were resuspended in HBS, pH 7.4. The monocytes were extracted from the washed PBMC by negative selection using a pan monocyte isolation kit (Miltenyi Biotec). Briefly, the cells were treated for 10 minutes with an FcR blocking reagent, then with a biotinylated antibody cocktail (specific to all white blood cells apart from monocytes) for 10 minutes. Streptavidin magnetic beads were added that bind to biotin on the antibodies and the cells were diluted and transferred to a 5-ml cylindrical tube. This was inserted into a magnet (eBioscience Affimetrix) and after 5 minutes the solution containing purified monocytes was decanted. The suspension was centrifuged, and the monocytes were resuspended in the desired volume. The purity was determined by flow cytometry using CD14 as a monocyte marker and CD41a as a platelet marker (Supplementary Fig. 2). Platelet contamination of monocyte preparations was generally 2%.

### Flow cytometry

Whole blood and isolated cell samples were stained for CD14 (APC, clone Tük4, Thermo Scientific), CD41a (FITC, clone HIP8, Thermo Scientific; and PeCy7, clone CB16, eBiosciences, CA, USA), CD62P (PE, clone 555524, and FITC, clone AK-4, BD Biosciences, CA, USA), CD142 (PE, clone HTF-1, Thermo Scientific; APC, clone HTF-1, eBiosciences; PE, clone NY2, BioLegend, CA, USA; FITC, clone #5, Sino Biological), activated integrin α_IIb_β_3_ (FITC, clone PAC-1, BD Biosciences), or CD15 (FITC, clone HI98, eBiosciences).

The samples were incubated for 5 minutes at room temperature. Isolated cell samples were fixed in 1% paraformaldehyde and whole blood samples in 1x Fix/Lyse (eBioscience) solution in ddH_2_O, to lyse red blood cells and fix the sample cells, for at least 15 minutes prior to data acquisition. 50 000 CD41a-positive events and at least 1000 CD14-positive events were collected per sample and analysed on a BD Accuri C6 flow cytometer (BD Biosciences). The accompanying software (BD Accuri version 1.0.264.21) was used for collecting and analysing the data. Fluorescence compensation was obtained using single stains of OneComp eBeads according to the manufacturer’s instructions (Thermo Scientific).

### Preparation of P-selectin-coated beads

Isolated monocytes were stimulated using P-selectin beads containing 6.25 μg/ml of P-selectin (Recombinant Human P-Selectin/CD62P Fc Chimera Protein; R&D Systems) coupled to 2 micrometre carboxyl polystyrene beads (Polysciences). P-selectin/Fc chimera protein was attached to 50 μl of Polybead® Carboxylate Microspheres, 2 μm (5.68 × 10^9^ particles per ml) using EDAC (carbodiimide; Sigma) dissolved in Coupling Buffer (50 mM MES, pH 5.2; 0.05% Proclin® 300) for covalent binding. The beads were resuspended in Wash/Storage Buffer (10 mM Tris, pH 8.0; 0.05% Bovine Serum Albumin [BSA], 0.05% Proclin 300) and stored at 4 °C. The P-selectin beads were warmed for 30 min at room temperature prior to use in the presence of 0.8% BSA to block non-specific binding. As a control, some beads were activated with EDAC in the absence of P-selectin. During preliminary experiments it was found that these beads also adhere to monocytes unless BSA is present in the Wash/Storage Buffer.

### Inhibitors, activators and blocking antibodies

All reagents were from Sigma unless otherwise stated. AR-C66096 and eptifibatide were from Tocris (Bristol, U.K.). PAR1-AP (SFLLRN-amide) and PAR4-AP (AYPGKF-amide) were from Bachem (Weil-am-Rhein, Germany). Cross-linked collegen-related peptide (CRP-XL) was synthesised by Arkadiusz Bonna, Department of Biochemistry. Function blocking antibodies were preservative-free. The antibody clones are listed in Supplementary Table 1.

### Data analysis

The number of repeats for experiments with whole blood and isolated cells refers to the number of experimental repeats, which took place on different days and using blood from different donors. GraphPad Prism v6 and FlowJo v10.5 were used for graphical presentation and statistical analysis. All graphs included represent mean + standard error of the mean (S.E.M.). One-way or two-way ANOVA, as appropriate, was used to determine statistical significance.

## Supplementary information

Supplementary figures 1-3

## Data Availability

The data are available from the corresponding author on reasonable request.
