# Supplementary material for: Platelet P-selectin triggers rapid surface exposure of tissue factor in monocytes
Source: Sci Rep. 2019 Sep 16;9:13397. doi: 10.1038/s41598-019-49635-7 (PMC6746844; doi:10.1038/s41598-019-49635-7)
Supplement: Supplementary file 1 — Supplementary figures 1-3 [file 41598_2019_49635_MOESM1_ESM.pdf]

Supplementary material for:

Platelet P-selectin triggers rapid surface exposure of tissue factor in monocytes

Ivelin I. Ivanov, Bonita H. R. Apta, Arkadiusz M. Bonna, Matthew T. Harper

Supplementary table 1: Function blocking antibodies used

Antibody	Clone	Manufacturer/Vendor
anti-ICAM1	HA58	eBiosciences
anti-ICAM2	CBR-IC2/2	Invitrogen
anti-CD40	S2C6	MABTech
anti-CD11b	M1/70	BioLegend
anti-CD18	TS1/18	BioLegend
anti-CD42b	HIP1	BioLegend
anti-CD62P (P-selectin)	AK4	BioLegend
anti-PSGL1	PL1	Ancell

Supplementary Figure 1:

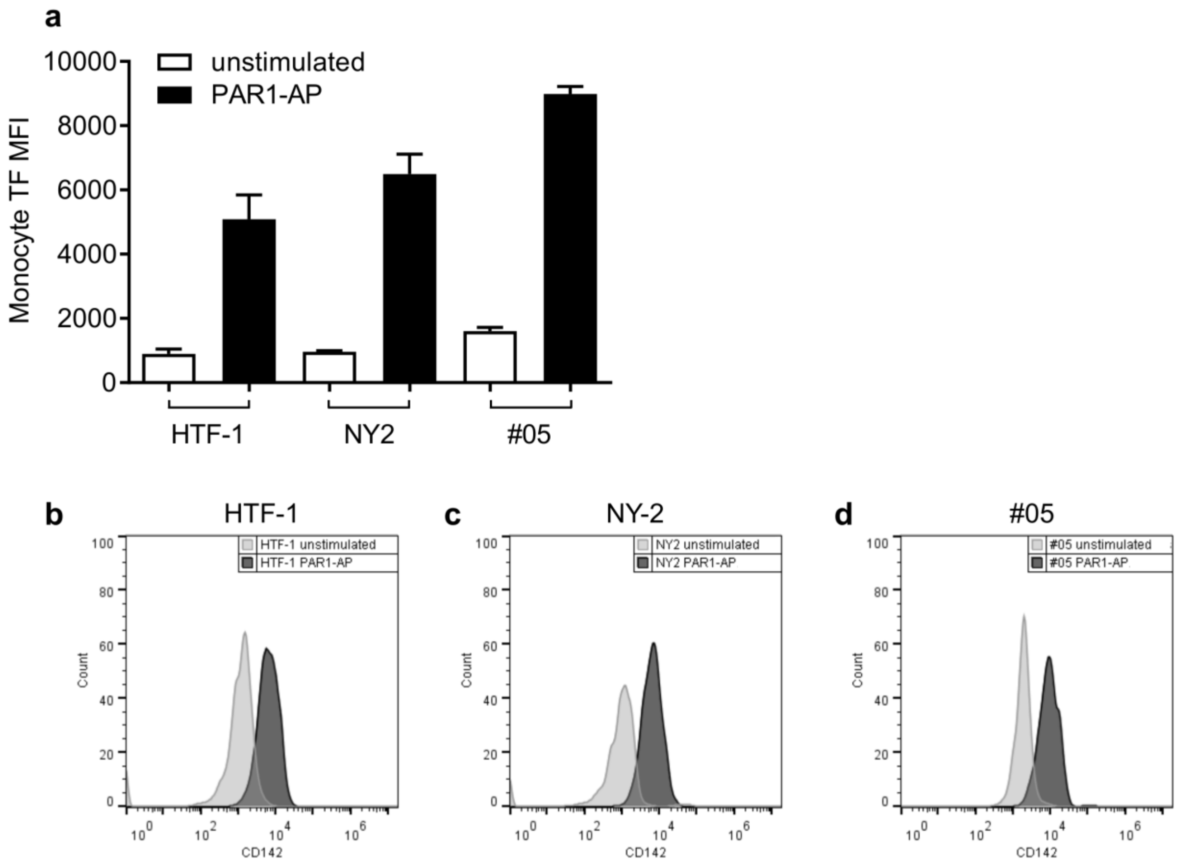

Supplementary Figure 1: Rapid monocyte TF exposure is detected by three different antibodies. (**a**) The increase in monocyte TF following PAR1-AP stimulation (10 µM, 25 min) of whole blood was detected using 3 different anti-TF antibody clones, HTF-1, NY2, #05. Data are mean + S.E.M. (n = 5). A representative histogram for each antibody clone is shown in (**b-d**).

Supplementary Figure 2:

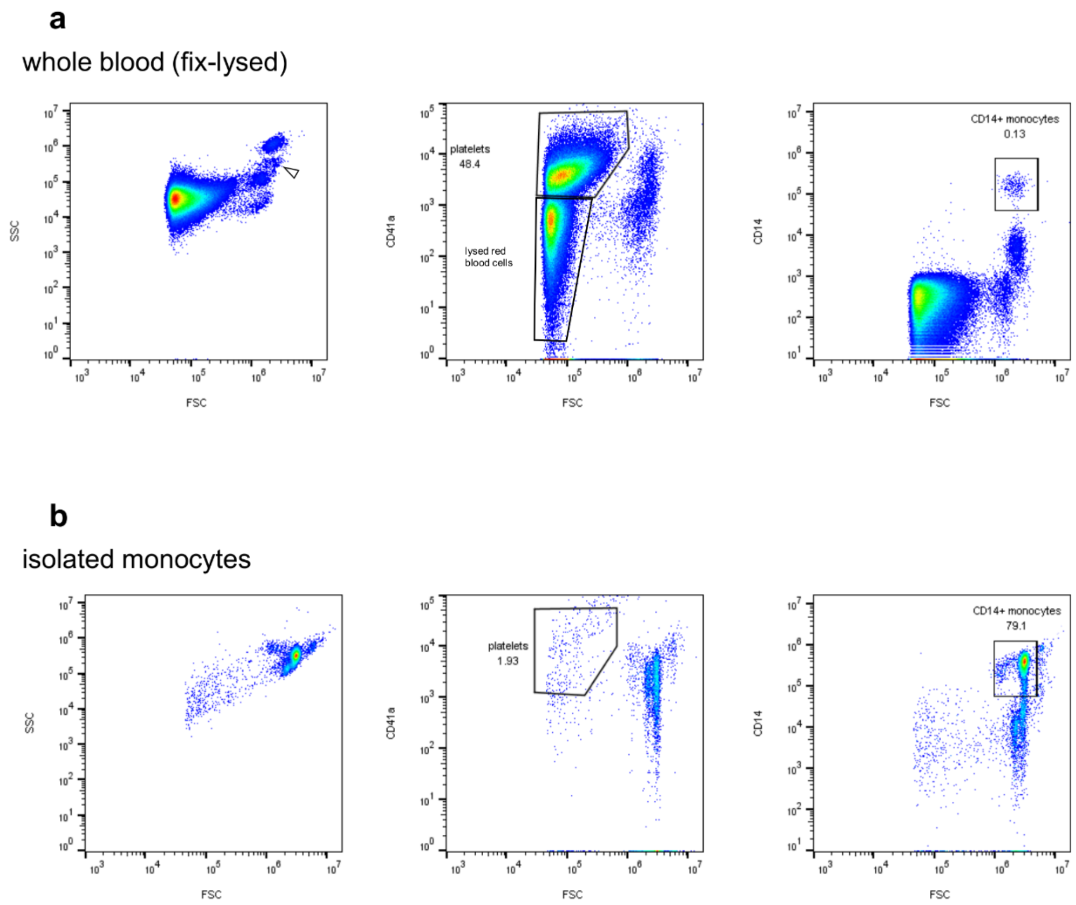

Supplementary Figure 2: Representative density plots of monocytes and platelets. Plots show the density of events on a colour scale from blue (low density) to red (high density). (**a**) Whole blood was stained with anti-CD14-APC and anti-CD41a-PerCP-Cy7, to detect monocytes and platelets, respectively. Samples were fixed in 1x Fix/Lyse. The FSC vs. SSC plot (left panel) shows a large heterogenous population of platelets and lysed red blood cells. These events could be distinguished by CD41a staining (middle panel). This provided an FSC vs CD41a region that is characteristic of platelets. Monocytes were detected by CD14 staining (right panel). The location of the CD14+ monocyte population in the left-hand panel is shown by the white arrowhead. (**b**) Monocytes were isolated from EDTA-anti-coagulated blood as described in the *Methods*. The FSC vs. SSC profile of this cell population is shown in the left-hand panel. Contaminating platelets were detected in the characteristic FSC vs CD41a region identified in (**a**) (middle panel). The CD14+ monocytes represent most of the cells (around 80 %).

Supplementary figure 3:

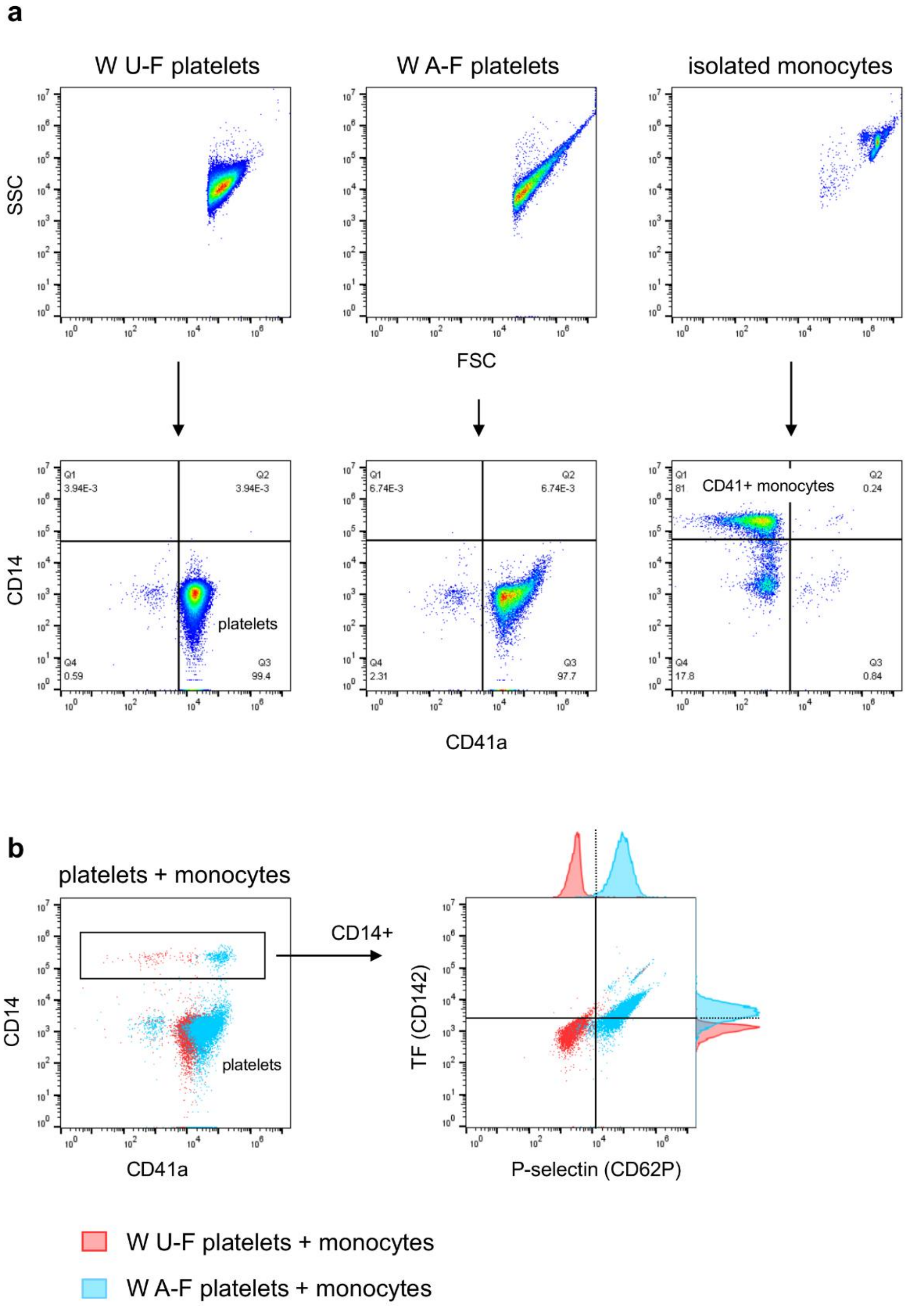

Supplementary Figure 3: Representative density plots of monocytes and fixed platelets. In (**a**), plots show the density of events on a colour scale from blue (low density) to red (high density). Washed unactivated-fixed (W U-F), washed activated-fixed (W A-F) and isolated monocytes are shown. Cells were stained with anti-CD14 and anti-CD41a antibodies to identify monocytes and platelets, respectively. Notably, in isolated platelets, CD14+ events were < 1%; in isolated monocytes, CD41a+ events were usually < 1%. (b) W U-F or W A-F platelets were added to isolated monocytes as described in the main text. Monocytes were identified as CD14+ events. (Note that

monocytes bind W A-F platelets.) TF (CD412) and P-selectin (CD62P) on these monocytes is shown. (The P-selectin derives from the activated platelets.) Monocytes incubated with W U-F platelets have little TF or P-selectin, whereas monocytes incubated with W A-F platelets have increased surface TF exposure and shown high expression of P-selectin on their surface. To aid interpretation, histograms of TF and P-selectin are shown at the sides of the plot. These plots are representative of 5 independent experiments. The quantification of these data is shown in Figure 3.
